# Risk factors and psychosocial characteristics of potential problematic and problematic internet use among adolescents: A cross-sectional study

**DOI:** 10.1186/1471-2458-11-595

**Published:** 2011-07-27

**Authors:** Georgios Kormas, Elena Critselis, Mari Janikian, Dimitrios Kafetzis, Artemis Tsitsika

**Affiliations:** 1 Adolescent Health Unit (A.H.U.), Second Department of Pediatrics, «P. & A. Kyriakou» Children's Hospital, National and Kapodistrian University of Athens School of Medicine, Greece; 2 Second Department of Pediatrics, «P. & A. Kyriakou» Children's Hospital, National and Kapodistrian University of Athens School of Medicine, Athens, Greece

**Keywords:** problematic internet use, adolescent, internet, psychosocial factors, behavior, addictive

## Abstract

**Background:**

Problematic internet use (PIU) is associated with a plethora of psychosocial adversities. The study objectives were to assess the determinants and psychosocial implications associated with potential PIU and PIU among adolescents.

**Methods:**

A cross-sectional study design was applied among a random sample (n = 866) of Greek adolescents (mean age: 14.7 years). Self-completed questionnaires, including internet use characteristics, Young Internet Addiction Test, and Strengths and Difficulties Questionnaire, were utilized to examine the study objectives.

**Results:**

Among the study population, the prevalence rates of potential PIU and PIU were 19.4% and 1.5%, respectively. Multinomial logistic regression indicated that male gender (Odds Ratio, OR: 2.01; 95% Confidence Interval, 95% CI: 1.35-3.00), as well as utilizing the internet for retrieving sexual information (OR: 2.52; 95% CI: 1.53-4.12), interactive game playing (OR: 1.85; 95% CI: 1.21-2.82), and socialization, including chat-room use (OR: 1.97; 95% CI: 1.36-2.86) and email (OR: 1.53; 95% CI: 1.05-2.24), were independently associated with potential PIU and PIU. Adolescents with potential PIU had an increased likelihood of concomitantly presenting with hyperactivity (OR: 4.39; 95% CI: 2.03-9.52) and conduct (OR: 2.56; 95% CI: 1.46-4.50) problems. Moreover, adolescent PIU was significantly associated with hyperactivity (OR: 9.96; 95% CI: 1.76-56.20) and conduct (OR: 8.39; 95% CI: 2.04-34.56) problems, as well as comprehensive psychosocial maladjustment (OR: 8.08; 95% CI: 1.44-45.34).

**Conclusions:**

The determinants of potential PIU and PIU include accessing the internet for the purposes of retrieving sexual information, game playing, and socialization. Furthermore, both potential PIU and PIU are adversely associated with notable behavioral and social maladjustment among adolescents.

## Background

Particularly among adolescents, the internet is observed to be increasingly adopted as a readily accessible means for information retrieval, entertainment, and socialization [1,2]. Excessive internet use may instigate potential adverse effects upon the psychosocial development of adolescents [3]. While both the adoption of excessive internet use, as well as related adverse psychosocial effects may be attributed to the compromised psychosocial wellbeing prior to the initiation of internet use [4], the likelihood of developing problematic patterns of behavior during adolescence is eminent [5,6]. Consequently, as adolescents allocate ever-increasing time periods for internet use, the risk for developing maladaptive internet use (MIU), including potential problematic internet use (PIU) and PIU, is inherent.

While PIU has received increasing research attention [7], a consistent definition of this construct has not been currently applied [8]. PIU has been proposed as a novel entity of dysfunctional behavioral patterns similar to those identified within the spectrum of impulse control disorders [9]. The proposed criteria for PIU initially included: (1) an uncontrollable use of the internet, (2) internet use which is markedly distressing, time consuming or resulting in social, occupational or financial difficulties; and (3) internet use not solely present during hypomanic or manic clinical episodes [10]. Hence, PIU is conceptualized as an individual's inability to control his/her use of the internet, thus causing marked distress and/or functional impairment [11,12]. Potential PIU is defined as internet use which fulfils some, but not all, of the above criteria [9,12,13].

Worldwide, the prevalence of PIU among adolescents and young adults has been observed to range between 0.9% [14] and 38% [15]. In particular, among European adolescents, the prevalence of PIU has been observed to range between 2% and 5.4% [6,16-18]. In Greece, the prevalence of PIU is observed to range between 1.0% [19] and 8.2% [20] among adolescents residing in rural and urban areas, respectively. Hence, PIU is markedly elevated among Greek adolescents as compared to their counterparts in other European countries.

Both excessive and PIU have been associated with a plethora of adverse psychosocial and mental health conditions. Specifically, the adoption of excessive internet use has been associated with social isolation [21] and related adversities [22]. Moreover, PIU has been associated with hostile behavioral patterns [23], impaired social skills [24], attention deficit hyperactivity disorder [14], and depression and/or suicidal ideation [25-27]. However, to date, evidence does not exist regarding the differential determinants and psychosocial implications of potential PIU and PIU among adolescents.

The primary objective of the present investigation is to evaluate the determinants of PIU and potential PIU among adolescents. The secondary objective is to evaluate the psychosocial characteristics and implications associated with PIU among the study population.

## Methods

### Study design and study population

A cross-sectional design was applied for the purposes of the study. All data were collected during two consecutive academic semesters (01/01/2007 - 01/01/2008). The study was approved by the Ethical Committees of both the "P. & A. Kyriakou" Children's Hospital in Athens, Greece, and the Hellenic Ministry of Education and Religious Affairs. Informed consent for study participation was required from the legal guardians of all eligible participants prior to the initiation of the study.

The source population for the present study consisted of a random cluster sample of 20 public junior high and high schools, stratified according to their locality and surrounding population density, in Athens, Greece. All students enrolled in Grades 9 and 10 of the selected schools were invited to participate in the study (n = 937). No exclusion criteria, including demographic and/or socioeconomic characteristics, for study participation were applied. The source population of the study consisted of 438 (46.7%) boys and 499 (53.3%) girls (overall mean age: 14.7 years). Seventy-one (7.6%) of the source population did not complete all components of the Young Internet Addiction Test and were thus excluded from all further statistical analyses. Hence, the response rate was 92.4% (N = 866).

### Data collection

Self-completed questionnaires were distributed to all study participants on-site at their respective schools. Study participants were requested to complete the questionnaire anonymously in order to minimize any potential reporting bias. The questionnaire consisted of 5 components: (1) demographic information; (2) history and average weekly hours of internet use; (3) location of internet access and scope of internet sites accessed; (4) the Young Internet Addiction Test; and (5) the Strengths and Difficulties Questionnaire.

Potential PIU and PIU were assessed through the application of the Young Internet Addiction Test (YIAT), as validated in the scientific literature [12,28-31]. The YIAT consists of 20 calibrated items for the evaluation of the degree of preoccupation, compulsive use, behavioral problems, emotional changes, and diminished functionality associated with internet use. Normal internet use, potential PIU, and PIU were defined according to the YIAT. Maladaptive internet use (MIU) was defined among those participants with either potential PIU or PIU [12].

In order to assess the history of internet use, the following cut-off values were applied: (1) novel users: 0-6 months; (2) recent users: 6-12 months; and (3) experienced users: > 12 months. The primary location of internet access assessed included internet access via (1) one's own home portal; (2) a friend's home portal; and, (3) internet café portal. The scope of internet sites accessed included: (1) e-mail correspondence; (2) access to the mass media (i.e. newspapers, journals, and periodicals); (3) chat-room use; (4) interactive game playing; (5) retrieval of information pertaining to work and education; and (6) retrieval of sexual education and information.

The Strengths and Difficulties (SDQ) Questionnaire was utilized to assess participants' emotional and psychosocial characteristics. The SDQ has served as a validated screening tool for evaluating the emotional and psychosocial difficulties of adolescents [32,33]. The five components of the SDQ and their respective scores are: (1) Emotional Symptoms Score (Normal: 0-5; Borderline: 6; Abnormal: 7-10); (2) Conduct Problems Score (Normal: 0-3; Borderline: 4; Abnormal: 5-10); (3) Hyperactivity Scale (Normal: 0-5; Borderline: 6; Abnormal: 7-10); (4) Peer Problems Scale (Normal: 0-3; Borderline: 4-5; Abnormal: 6-10); and (5) Prosocial Scale (Normal: 6-10; Borderline: 5; Abnormal: 0-4). With the exclusion of the Prosocial Scale, the sum of the remaining SDQ component scores was derived to generate the Total Difficulties Score (Normal: 0-15; Borderline: 16-19; Abnormal: 20-40).

### Statistical analysis

Student's t-test for independent samples was applied to compare the mean values of continuous variables and the chi-squared test was used to compare the differences in proportions of categorical variables between groups, respectively. Fisher's Exact Test was applied instead when at least one comparison group consisted of ≤ 5 adolescents. Age and gender adjusted odds ratios (AOR) and 95% Confidence Intervals (95% CI) were calculated to assess the likelihood of the characteristics of internet use, as well as SDQ component and total scores, between the study groups. Stepwise multinomial logistic regression analyses were applied in order to evaluate the determinants of potential PIU and PIU, as compared to normal internet use. The independent variables included in the multivariate regression models included the locations of internet access and scope of internet sites utilized. A p-value (*p*) of ≤ 0.05 was the criterion for significance. Statistical analyses were conducted with the application of the SAS version 9.0 (SAS Institute Inc., USA) software package.

## Results

### Overall maladaptive internet use (MIU)

Among the study population (n = 866), the prevalence rate of maladaptive internet use (MIU) was 20.9% (n = 181). The mean age (± standard deviation, SD) of adolescents with MIU did not significantly differ from that of their normal internet user counterparts (14.8 ± 0.6 years vs. 14.8 ± 0.6 years, p = 0.838). However, adolescents with MIU were 2.91 (95% Confidence Interval, 95% CI: 2.07-4.13) times more likely to be male as compared to normal internet users. Moreover, the proportion of adolescents with MIU reporting poor academic performance was greater than that among normal internet users (Table 1).

**Table 1:** Characteristics of the study population according to the degree of maladaptive internet use (n = 866).

	Overall maladaptive internet use (n = 181)		Problematic internet use (n = 13)		Potential problematic internet use (n = 168)		Normal internet use (n = 685)
	n (%)	p-value	n (%)	p-value	n (%)	p-value	n (%)
**Age ***mean years ± SD*	14.8 ± 0.6	0.838	14.6 ± 0.4	0.268†	14.8 ± 0.6	0.625†	14.8 ± 0.6
**Gender**							
Male	122 (67.4)	Referent	11 (84.6)	Referent	111 (66.1)	Referent	284 (41.5)
Female	59 (32.6)	*< 0.0001*	2 (15.4)	*0.002*‡	57 (33.9)	*< 0.0001*‡	401 (58.5)
**Academic performance**							
Average	82 (45.3)	Referent	5 (38.5)	Referent	77 (45.8)	Referent	193 (28.2)
Good	74 (40.9)	0.003	7 (53.8)	0.229§	65 (38.7)	*0.002*‡	292 (42.6)
Excellent	27 (14.9)	*< 0.0001*	1 (7.7)	0.090§	26 (15.5)	*< 0.0001*‡	200 (29.2)
**Internet use experience**							
0-6 months	33 (18.2)	Referent	1 (7.7)	Referent	32 (19.0)	Referent	289 (42.2)
6-12 months	24 (13.2)	*0.0006*	0 (0.0)	0.788§	24 (14.3)	*< 0.0001*‡	79 (11.5)
> 12 months	124 (68.5)	*< 0.0001*	12 (92.3)	*0.004*‡	112 (66.7)	*< 0.0001*‡	317 (46.3)

† Student's t-test p-value

‡ Chi-squared p-value

§ Fischer's exact test p-value

With respect to the locations of internet access, adolescents with MIU were significantly more likely to access the internet via internet café portals and their own home portal as compared to normal internet users as shown in Table 2. Moreover, with regard to the scope of internet sites accessed, adolescents with MIU were approximately twice as likely to access the internet for the purposes of chat-room use and interactive game-playing. Adolescents with MIU were also 2.70 (95% CI: 1.66-4.38) times more likely to access the internet for the purposes of sexual information as compared to their normal internet user counterparts. Finally, adolescents with MIU were significantly less likely to access the internet for the purposes of education.

**Table 2:** Likelihood of locations and scope of internet sites accessed according to the degree of maladaptive internet use.

	Overall maladaptive internet use (n = 181)		Problematic internet use (n = 13)		Potential problematic internet use (n = 168)		Normal internet use (n = 685)
	n (%)	AOR* (95% CI)	n (%)	AOR* (95% CI)	n (%)	AOR* (95% CI)	n (%)
**Locations of internet access**							
Own house	142 (78.4)	*1.76 (1.18-2.62)*	11 (84.6)	2.76 (0.60-12.68)	131 (78.0)	*1.71 (1.14-2.56)*	452 (66.0)
Friend's house	38 (21.0)	0.86 (0.57-1.30)	3 (23.9)	1.00 (0.27-3.72)	35 (20.8)	0.86 (0.56-1.30)	163 (23.8)
Internet café	107 (57.5)	*1.90 (1.34-2.70)*	9 (69.2)	2.76 (0.82-9.31)	95 (56.5)	*1.86 (1.29-2.66)*	237 (34.6)
**Scope of internet sites used**							
E-mail	104 (57.4)	*1.83 (1.30-2.58)*	9 (69.2)	2.94 (0.88-9.76)	95 (56.5)	*1.76 (1.24-2.50)*	285 (41.6)
Mass media	30 (16.6)	1.00 (0.63-1.57)	0 (0.0)	NC	30 (17.8)	1.08 (0.68-1.69)	114 (16.6)
Chat room	101 (55.8)	*2.28 (1.62-3.21)*	9 (69.2)	*3.73 (1.12-12.38)*	92 (54.8)	*2.19 (1.54-3.12)*	230 (33.6)
Games	137 (75.7)	*1.95 (1.31-2.90)*	12 (92.3)	5.78 (0.72-46.32)	125 (74.4)	*1.86 (1.24-2.78)*	364 (53.1)
Education	38 (21.0)	*0.61 (0.40-0.91)*	4 (30.8)	1.12 (0.33-3.80)	34 (20.2)	*0.57 (0.38-0.87)*	232 (33.9)
Sexual information	38 (21.0)	*2.70 (1.66-4.38)*	6 (46.2)	*6.77 (2.09-21.97)*	32 (19.0)	*2.43 (1.46-4.02)*	48 (7.0)

*AOR: Adjusted Odds Ratio for age and sex

95% CI: 95% Confidence Interval

NC: Not computable

The comparison of the emotional and psychosocial characteristics between adolescents with MIU and normal internet use are shown in Table 3. Adolescents with MIU were in excess of two times more likely to have an Abnormal Conduct Problems score and of four times more likely to have an Abnormal Hyperactivity Score, respectively. Hence, MIU was associated with both notable behavioral maladjustment and hyperactivity problems among adolescents. Furthermore, adolescents with MIU were approximately three times more likely to report an Abnormal Total SDQ score as compared to normal internet users. Therefore, MIU was associated with comprehensive emotional and psychosocial maladjustment among adolescents.

**Table 3:** Likelihood of Strengths and Difficulties Questionnaire according to the degree of maladaptive internet use.

	Overall maladaptive internet use (n = 181)		Problematic internet use (n = 13)		Potential problematic internet use (n = 168)		Normal internet use (n = 685)
	n (%)	AOR* (95% CI)	n (%)	AOR* (95% CI)	n (%)	AOR* (95% CI)	n (%)
**Emotional**							
Borderline	19 (10.5)	1.74 (0.97-3.11)	1 (7.7)	1.95 (0.23-16.76)	18 (10.7)	1.73 (0.96-3.12)	55 (8.0)
Abnormal	11 (6.1)	0.93 (0.46-1.85)	2 (15.4)	3.15 (0.61-16.30)	9 (5.4)	0.78 (0.37-1.64)	69 (10.1)
**Conduct problems**							
Borderline	49 (27.1)	*4.35 (2.78-6.82)*	3 (23.1)	*5.60 (1.26-24.84)*	46 (27.4)	*4.29 (2.72-6.76)*	59 (8.6)
Abnormal	26 (14.4)	*2.82 (1.65-4.84)*	4 (30.8)	*8.39 (2.04-34.56)*	22 (13.1)	*2.56 (1.46-4.50)*	47 (6.9)
**Hyperactivity**							
Borderline	20 (11.0)	*1.99 (1.12-3.53)*	2 (15.4)	3.26 (0.64-16.69)	18 (10.7)	*1.91 (1.05-3.46)*	46 (6.7)
Abnormal	16 (8.8)	*4.87 (2.29-10.35)*	2 (15.4)	*9.96 (1.76-56.20)*	14 (8.3)	*4.39 (2.03-9.52)*	16 (2.3)
**Peer problems**							
Borderline	32 (17.7)	1.46 (0.92-2.31)	1 (7.7)	0.55 (0.07-4.39)	31 (18.4)	1.54 (0.97-2.46)	86 (12.6)
Abnormal	8 (4.4)	2.48 (0.94-6.50)	1 (7.7)	5.52 (0.54-47.12)	7 (4.2)	2.32 (0.82-6.05)	11 (1.6)
**Prosocial**							
Borderline	17 (9.4)	1.12 (0.61-2.04)	2 (15.4)	1.73 (0.36-8.37)	15 (8.9)	1.08 (0.57-2.02)	46 (6.7)
Abnormal	14 (7.7)	1.11 (0.58-2.13)	2 (15.4)	0.84 (0.10-6.95)	13 (7.7)	1.12 (0.57-2.19)	39 (5.7)
***Total SDQ Score***							
Borderline	30 (16.6)	*2.27 (1.38-3.72)*	23 (7.3)	3.47 (0.66-18.26)	28 (16.7)	*2.25 (1.36-3.72)*	71 (10.4)
Abnormal	11 (6.1)	*2.96 (1.33-6.60)*	8 (2.5)	*8.08 (1.44-45.34)*	9 (5.4)	*2.55 (1.09-5.94)*	19 (2.8)

*AOR: Adjusted Odds Ratio for age and sex, 95% CI: 95% Confidence Interval

### Potential problematic internet use (PIU)

Among the study population the prevalence of rate of potential PIU (mean YIAT score ± standard deviation, SD: 48.9 ± 7.2) was 19.4% (n = 168). Adolescents with potential PIU were 2.77 (95% CI: 1.92-3.85) times more likely to be male. While adolescents with potential PIU did not differ from their normal internet user counterparts with respect to age, they were more than twice as likely to be either recent (Odds ratio, OR: 2.56; 95% CI: 1.40-4.65) or experienced (OR: 2.78; 95% CI: 1.80-4.28) internet users. In addition, poor academic performance was reported more frequently among adolescents with potential PIU as compared to their normal internet user peers (Table 1).

Adolescents with potential PIU were significantly more likely to utilize their own home portal and internet café portals as compared to their normal internet user counterparts (Table 2). Furthermore, with regard to the scope of internet sites accessed, the likelihood of utilizing the internet for the purposes of retrieving sexual information and/or content was 2.43 times greater among adolescents with potential PIU (Table 2). In addition, adolescents with potential PIU were approximately twice as likely to utilize the internet for the purposes of socialization and communication, such as chat-rooms and email. Moreover, the likelihood utilizing the internet for game-playing was 1.86 times greater among this population group as compared to normal internet users. It is noteworthy, though, that among the adolescent population evaluated potential PIU was inversely associated with utilizing the internet for educational purposes.

Potential PIU among adolescents was associated with an increased likelihood of Abnormal Hyperactivity and Conduct Problems scores as compared to their normal internet user counterparts (Table 3). Even so, adolescents with potential PIU were not observed to differ with respect to their emotional and social spheres from their normal internet user counterparts. However, adolescents with potential PIU were in excess of two times more likely to have comprehensive psychosocial maladjustment as compared to their normal internet user peers.

### Problematic internet use (PIU)

The prevalence rate of PIU (mean YIAT score ± SD: 79.3 ± 7.5) among the study population was 1.5% (n = 13). Adolescents with PIU were in excess of seven times more likely than their normal internet user counterparts to be male. In addition, adolescents with PIU were more than eight times more likely to report > 12 months of internet use (Table 1).

Adolescents with PIU more frequently utilized internet café portals as compared to their normal internet user counterparts (p = 0.018). Moreover, adolescent PIU was significantly associated with accessing the internet for the purposes of retrieving sexual information and/or sexual content and chat room use (Table 2). It is noteworthy that while the majority of adolescents with PIU utilized the medium for the purposes of interactive game-playing, such use did not significantly differ from that of their normal internet user counterparts (Table 2).

Adolescents with PIU were observed to have an enhanced likelihood for concomitantly presenting with hyperactivity and conduct problems scores (Table 3). Specifically, according to the SDQ component scores, the odds of abnormal hyperactivity and conduct problems scores were approximately ten and eight times greater, respectively, among adolescents with PIU as compared to normal internet users. Moreover, adolescent PIU was not significantly associated with emotional and social maladjustment. However, adolescents with PIU were approximately eight times more likely to have comprehensive psychosocial maladjustment, as indicated by the total SDQ score.

### Determinants of potential PIU and PIU

The multinomial logistic regression analysis (Table 4) indicated that male gender, utilizing the internet for retrieving sexual information, interactive game playing, and socialization, including chat-room use and email, were independently associated with potential PIU and PIU.

**Table 4:** Factors independently associated with maladaptive internet use.

	Intercept (β)	Standard Error (SE)	Wald p-value	AOR* (95% CI)
Male gender	0.70	0.20	*0.006*	*2.01 (1.35-3.00)*
Utilizing the internet for retrieving sexual information	0.92	0.25	*0.0003*	*2.52 (1.53-4.12)*
Utilizing the internet for socialization and communication:				
a) Chat rooms	0.68	0.19	*0.0004*	*1.97 (1.36-2.86)*
b) E-mail	0.43	0.19	*0.027*	*1.53 (1.05-2.24)*
Utilizing the internet for interactive game-playing	0.61	0.22	*0.005*	*1.85 (1.21-2.82)*
Utilizing the internet for educational purposes	-0.68	0.23	*0.002*	*0.50 (0.32-0.79)*

*AOR: Adjusted Odds Ratio for age and sex, 95% CI: 95% Confidence Interval

## Discussion

The present study is the first of its kind to assess the internet use characteristics associated with both potential PIU and PIU among adolescents. Moreover, it is also the first of its kind to evaluate both the solitary and differential psychosocial implications associated with PIU among adolescents according to the degree of maladaptive behavioral patterns adopted.

The study findings indicated that the prevalence rate of PIU among adolescents was 1.5%. The observed prevalence rate is within the lower range of those reported both in Greek rural areas and in other European countries [6,16,18,20,34] and may be attributed to the limited penetration of computer/internet access among urban Greek youth [35]. However, marked international variances regarding the prevalence rates of PIU may also be attributed to a measurement bias incurred by a lack of international consistency regarding both the definition and assessment of PIU [8]. Furthermore, among the study population examined approximately one fifth (19.4%) of adolescents were identified with potential PIU. It is upheld that such internet users are at an enhanced risk for developing PIU.

The majority of adolescents with either potential PIU or PIU were male. Similar gender differences regarding the frequency and nature of internet use have been previously reported [36]. The gender differences observed may be attributed to the potential confounding effect of the differential frequency of internet utilization between genders. Specifically, since adolescent boys utilize the internet both more frequently and extensively than adolescent girls [19], the average weekly hours of internet utilization may serve as a potential confounder for the development of PIU, particularly among adolescent males.

The study findings indicated that potential PIU and PIU were independently associated with utilizing the internet for the purposes of retrieving sexual information, socialization, and entertainment, including interactive game-playing. Moreover, it is noteworthy that potential PIU was inversely associated with utilizing the internet for educational purposes. Previous reports indicate that more than one quarter of frequent internet users utilize the internet for accessing sexual information and education [19,37,38]. Both frequent internet use and accessing the internet for the purposes of sexual education have been found to be significant predictors of pornographic internet site use [39,40] and consequent PIU [41]. Hence, it is proposed that PIU may develop and/or manifest secondary to the specific content of internet sites accessed, rather than to the internet per se.

With regard to the psychosocial implications of PIU, including potential PIU and PIU, the study findings indicated that such behaviors are associated with an enhanced likelihood of hyperactivity and conduct problems. It is important to note, though, that while the likelihood of conduct problems was more than three times greater among adolescents with PIU as compared to those with potential PIU, the odds of hyperactivity problems was approximately two times greater, respectively. To date, similar findings regarding the likelihood of hyperactivity and conduct problems among adolescent with potential PIU have not been reported.

The evidence provided regarding the concomitant occurrence of conduct problems and PIU corroborates with related findings in the literature indicating that adolescents with PIU tend to be lonelier [42] and adopt more aggressive behaviors [43]. Moreover, previous findings have indicated that conduct problems among youth with PIU may be proximally associated with increased social isolation and impaired communication skills [24]. However, the present study findings indicated that adolescents with either potential PIU or PIU did not present with deterred peer relations and/or social skills. It is posited that adolescents may counteract their possible real world social isolation with increased use of cyber communication and socialization platforms, thus retaining social networks through the internet medium.

The present study indicated that neither potential PIU nor PIU among adolescents were significantly associated with emotional maladjustment. These findings contrast those established in the literature indicating that emotional symptoms, such as depressive and anxiety symptoms, have been associated with PIU [9,44-47]. It is posited that the emotional adjustment among adolescents with either potential PIU or PIU may be secondary to a potential population bias introduced by the study sampling applied. Specifically, due to the fact that the study population was recruited from students attending public junior high and high schools, those adolescents with severely impaired functionality, including both severely deterred academic performance to the extent of exclusion and/or expulsion from academic attendance and activities, may have not been included in the study population.

Furthermore, the present study indicated that adolescents with potential PIU or PIU were more than two and eight times, respectively, as likely to have global emotional and psychosocial maladjustment, as assessed by the total SDQ score. A correlation between PIU and compromised psychological well-being has been previously documented [42,48]. However, differential psychosocial impacts according to the degree of PIU have not been reported. Thus, the present study provides evidence that while adolescents with PIU exhibit marked behavioral and psychosocial maladjustment, adolescents with potential PIU also have a limited, albeit notable, increased risk for manifesting comprehensive emotional and psychosocial impairments.

Therefore, the study findings indicate that both potential PIU and PIU are associated with notable emotional and psychosocial maladjustment among adolescents. It is upheld that such internet behaviors may constitute an escape mechanism for adolescents to temporarily relieve and/or escape from emotional and behavioral difficulties [49]. Therefore, adolescents may use the internet excessively in order to cope with emotional turmoil. Concurrently, PIU has been observed to lead to unsuccessful life-coping mechanisms [50]. It is posited that poorly adjusted adolescents, may suffer more harmful effects following PIU, thus creating a vicious gyration centered upon internet usage and psychosocial maladjustment. Consequently, PIU may compound pre-existing psychosocial symptomatology present among adolescents.

The strengths of the present study include that it is the first of its kind conducted in order to assess both the determinants and psychosocial effects of potential PIU and PIU among adolescents in Greece. Due to the random sampling applied for the selection of the study population, it is upheld that the potential introduction of a selection bias was deterred. The limitations of the study include its inability to decipher the etiological association between PIU and the psychosocial characteristics of adolescents due to the cross-sectional study design applied. In addition, psychiatric conditions and other risk factors could not be assessed in relation to the occurrence and development of maladaptive internet use. Finally, since adolescents belonging to the same class and/or school may potentially utilize internet applications with each other, a clustering effect upon the association between the use of cyber social networking, as well as gaming, in relation to maladaptive internet use may have been introduced. Since a stratified cluster sample was used for the present investigation, both the standard errors and confidence intervals reported may be an underestimation of their true magnitude. Further prospective investigations are necessary in order to assess both such clustering effects and whether the psychosocial characteristics observed among adolescents with potential PIU may constitute potential risk factors for the consequent development of PIU.

## Conclusions

Among adolescents the prevalence rates of potential PIU and PIU were observed to be 19.4% and 1.5%, respectively. Multinomial logistic regression indicated that potential PIU and PIU were significantly associated with male gender, as well as utilizing the internet for retrieving sexual information, interactive game playing, and socialization, including chat-room use and email. Adolescents with potential PIU had an increased likelihood of concomitantly presenting with hyperactivity and conduct problems. Moreover, adolescent PIU was significantly associated with hyperactivity and conduct problems, as well as comprehensive psychosocial maladjustment. Therefore, the determinants of potential PIU and PIU include accessing the internet for the purposes of retrieving sexual information, game playing, and socialization. Furthermore, both potential PIU and PIU adversely associated with notable behavioral and social maladjustment among adolescents.

## Competing interests

The authors declare that they have no competing interests.

## Authors' contributions

GK participated in the conception and design, acquisition of data, and manuscript composition. EC performed the statistical analysis and interpretation of data, and participated in the composition and critical revision of the manuscript. MJ participated in the composition and critical revision of the manuscript. DK helped critically revise the manuscript for intellectual content. AT participated in the study design and coordination. All authors read and approved the final manuscript.

## Pre-publication history

The pre-publication history for this paper can be accessed here:

http://www.biomedcentral.com/1471-2458/11/595/prepub
